# TLR4 activation by lysozyme induces pain without inflammation

**DOI:** 10.3389/fimmu.2023.1065226

**Published:** 2023-05-01

**Authors:** Saurabh Yadav, Amrita Singh, Ravi Kant, Avadhesha Surolia

**Affiliations:** ^1^ Molecular Biophysics Unit, Indian Institute of Science, Bangalore, India; ^2^ Institute of Science, Nirma University, Ahmedabad, India

**Keywords:** lysozyme, TLR4 – toll-like receptor 4, pain, inflammation, MyD88, LPS -lipopolysaccharide, TRIF, PNS -peripheral nervous system

## Abstract

Mostly, pain has been studied in association with inflammation, until recent studies which indicate that during bacterial infections, pain mechanisms could be independent of the inflammation. Chronic pain can sustain long after the healing from the injury, even in the absence of any visible inflammation. However, the mechanism behind this is not known. We tested inflammation in lysozyme-injected mice foot paw. Interestingly, we observed no inflammation in mice foot paw. Yet, lysozyme injections induced pain in these mice. Lysozyme induces pain in a TLR4-dependent manner and TLR4 activation by its ligands such as LPS leads to inflammatory response. We compared the intracellular signaling of MyD88 and TRIF pathways upon TLR4 activation by lysozyme and LPS to understand the underlying mechanism behind the absence of an inflammatory response upon lysozyme treatment. We observed a TLR4 induced selective TRIF and not MyD88 pathway activation upon lysozyme treatment. This is unlike any other previously known endogenous TLR4 activators. A selective activation of TRIF pathway by lysozyme induces weak inflammatory cytokine response devoid of inflammation. However, lysozyme activates glutamate oxaloacetate transaminase-2 (GOT2) in neurons in a TRIF-dependent manner, resulting in enhanced glutamate response. We propose that this enhanced glutaminergic response could lead to neuronal activation resulting in pain sensation upon lysozyme injections. Collectively we identify that TLR4 activation by lysozyme can induce pain in absence of a significant inflammation. Also, unlike other known TLR4 endogenous activators, lysozyme does not activate MyD88 signaling. These findings uncover a mechanism of selective activation of TRIF pathway by TLR4. This selective TRIF activation induces pain with negligible inflammation, constituting a chronic pain homeostatic mechanism.

## Introduction

Inflammation is a defence response of the body towards infection or injury which localizes and eliminates the injurious agents and promotes clearance of cellular debris leading to tissue repair and regeneration. Pain is one of the cardinal features of inflammation (1, 2). Acute inflammatory pain is a warning mechanism against a hazardous stimulus and thus helps in preventing further damage to an injured area during the healing process (3). Injury to the nervous tissues and associated neuroinflammatory condition leads to neuropathic pain, a major debilitating condition affecting about 10% adults worldwide (4).

Heightened expression of various immune mediators (viz. chemokines, cytokines, growth factors etc.) during tissue inflammation results in enhanced pain sensitization of the injured area (5). Consequently, role of immune mediators in genesis of pain is an active area of research (1, 6). Immune modulation of nociceptors during inflammatory or neuroinflammatory conditions lead to reduced thresholds for nociceptor firing resulting in pain sensitivity (hyperalgesia) (5, 7). Pain tends to reduce with resolution of inflammation, indicating a central role of immune mediators in neuronal sensitization during inflammation (2, 8). Thus, acute pain, which is often associated with the inflammatory conditions and reduces with the resolution of tissue immune response, is not a problem in itself. Chronic pain, on the other hand, is a painful condition persisting even after the tissue inflammatory response is subdued and hence is a disease condition (9, 10). Therefore, understanding the mediators and mechanisms associated with heightened pain in the absence of a robust inflammatory response is critical for our understanding about chronic pain and its management.

Studies suggest that pain is independent of the inflammatory stimuli such as bacterial load during infection and hence indicate that its pathophysiology could be independent of the concurrent inflammatory response (11). Also, chronic neuropathic pain often outlasts inflammation and the classical non-steroid anti-inflammatory drugs are not very effective against chronic neuropathic pain (12). These observations indicate that pain can be independent of inflammation, and pain can sustain itself in the absence of inflammation. Peripheral inputs are key to the development and maintenance of central sensitization associated with the neuropathic pain (13). Yet, the mechanistic details as to how chronic pain is maintained in absence of a robust inflammatory response especially after sterile injuries are missing.

Neuro-immune and neuro-glial crosstalk are pivotal in the pathogenesis of chronic pain during neuroinflammation. Toll like receptors (TLRs) play a key role in the neuro-immune and neuro-glial interactions during neuropathological conditions including chronic pain (14). Out of all the TLRs, TLR4 is of great interest as it is expressed by glial cells, immune cells and by primary sensory neurons of the dorsal root ganglion of the peripheral nervous system which are involved in pain pathology (15–18). While TLR4-mediated activation of glial cells, and subsequent release of immune mediators (cytokines) modulating neuronal physiology is well established, the expression and role of neuronal innate immune receptor(s) signaling during neuropathologies remain poorly understood. Moreover, whether TLR4 activation by different ligands affects host cells physiology differently, is also unclear. Thus, unlike glial cells, how neuronal TLR4 activation occurs and what role it plays during neuropathologic pain homeostasis remains unknown. Here we explored the effects of lysozyme-mediated neuronal TLR4 activation on neuroinflammation and pain homeostasis.

## Materials and methods

Lysozyme (Sigma #L6394), LPS (Sigma #L2630), Glutamate assay kit (Sigma #MAK004) and Minocycline were purchased from Sigma. TLR4 (Abcam #ab22048), PGP9.5 (Abcam #ab108986), GOT (Abcam #ab221939) and Glutaminase (Abcam #ab156876) antibodies were purchased from Abcam. NFκB p65 (CST #8242), Glutamate dehydrogenase (CST #80063), GOT1 (CST #34423), and GOT2 (CST #71692) antibodies were purchased from Cell Signaling Technology. PEPinh MyD, PEPinh TRIF were purchased from Invivogen. Cu-CPT22 (#4884), TAK-242 (#6587), and Dynasore (#2897) were purchased from Tocris. TRIF siRNA (Thermo, #4457308), GOT2 siRNA (Thermo, #4457308), HMGB1 (Thermo #34-8401-82), IFNα mouse ELISA kit (Thermo #BMS6027) and IL-10 mouse ELISA kit (Thermo #BMS614) were purchased from ThermoFisher Scientific. IFN-β ELISA kit (PBL Assay Science #424001) was purchased from PBL Assay Science and mouse inflammatory cytokines ELISA array kit (Qiagen #336161) was purchased from Qiagen. Cell culture media and reagents were purchased from Thermo (Invitrogen). All other reagents were purchased from Sigma until stated otherwise.

### Animals and experimental protocol

This study was carried out in strict accordance with the guidelines of International Association for the Study of Pain and the Institutional Animal Ethics Committee. C57BL/6J mice, C3H/HeJ (TLR4 inactive mutant) mice and B6.129-Tlr2^tm1Kir^/J (TLR2 knockout) mice were provided by the animal facility of the Indian Institute of Science, Bangalore, India. MyD88 knockout mice were provided by the animal facility of the National Institute of Immunology, New Delhi, India. Animals of either sex, studied at the age of 6-10 weeks, were kept at constant temperature (25°C) in a standard light/dark cycle (12 Hrs/12 Hrs). Four mice were housed per cage with free access to food and water. All the experiments were performed in groups which were age and sex matched.

### Intrathecal siRNA injections

*In-vivo* ready TRIF, and GOT2 -siRNAs, and a scrambled siRNA were dissolved in aCSF and injected (400 µg/animal) using 25 µl 30-gauge Hamilton syringe, between 5^th^ and 6^th^ lumbar vertebrae, daily for 3 days prior to lysozyme injection. All intrathecal injections were performed under isoflurane anaesthesia.

### Foot paw injections

Animals of same age and groups were divided into groups of approximately same numbers. Stocks of LPS (10μg/50μl) and HMGB1 (100μg/50μl) were prepared in DMSO solvent, and Lysozyme (100μg/50μl) was dissolved in PBS. LPS, HMGB1, Lyz and control vehicles were injected into the intraplantar surface of mice foot paw using a 100µl Hamilton syringe. Similarly the control groups were injected with same volume of vehicle solutions. Same method was used to inject different compounds into the mice foot paw. The animals were then tested for mechanical allodynia using *Von Frey*.

### Behavioral testing

Animals were placed on a wire mesh platform and were covered with a clear cage. They were allowed to acclimate for 30 min before injections. *Von Frey* filaments (IITC) were applied perpendicularly to the planter surface of the hind paw (ipsilateral to the foot paw injections). Mechanical sensitivity was assessed by sequentially increasing and decreasing the stimulus. A crisp withdrawal of the paw was taken as a positive response. For foot paw inflammation, a digital micrometer (Mitutoyo) was used to measure thickness of the plantar area before and after time points post injections. Change in thickness was calculated as differences from before measurements.

### Evans blue assay

To assess inflammation-associated vascular permeability EB assay was performed. Briefly, 6-8 week-old mice were infused with 50μl of EB (Sigma #E2129) (25mg/ml) intravenously. EB was allowed to circulate for 15 minutes. Subsequently, foot pads of hind paws were injected either with LPS (10μg/50μl) or Lysozyme (100μg/50μl). 30 minutes later mice were perfused with ice-cold PBS and paw tissue were harvested and allowed to dry in the oven at 56°C for two days. EB was extracted using formamide. A standard plot for EB was prepared and the amount of EB released was estimated.

### Immunofluorescence

Mice used for immunofluorescence were euthanized with a high dose of isoflurane and transcardially perfused with 4% PFA. The foot paw glabrous skin tissue was dissected removing the ligaments. The samples were then fixed with 4% PFA at 4°C for 2 Hrs. Tissues were cryo-protected in 30% sucrose in PBS (4% overnight) before freezing. Frozen glabrous skin were cut (20µm slices) on a Leica CM1950 cryostat. The tissues were then blocked with 3% BSA in PBS for 1 Hr at room temperature followed by incubation with the primary antibodies. For fluorescence immunostaining, the following primary antibodies were used: PGP 9.5 (1:500), and TLR4 (1:400). The sections were then washed with PBS thrice, incubated with Alexa-fluor 488 lebeled anti-rabbit secondary antibody (1:600) and Alexa-fluor 598 lebeled anti-mouse secondary antibody (1:600) in dark at room temperature for 2 Hrs. Subsequently, samples were washed four times with PBS, mounted using ProLong Gold antifade reagent (Thermo #P36935) and images were acquired using a Ziess LSM 510 Meta confocal microscope under identical settings.

### Protein preparation from cell and tissue lysates and western blotting

THP-1 cells were cultured in Roswell Park Memorial Institute (RPMI) 1640 Medium, supplemented with 10% fetal bovine serum (FBS) and 1% Penicillin/Streptomycin. After reaching confluency, cells were seeded at 1 × 10^5^ cells/mL density and stimulated with 10 ng/mL PMA for 24 Hrs to differentiate into macrophages. Then, they were subsequently maintained in PMA-free serum-containing RPMI 1640 medium for 24 Hrs. SH-SY5Y human neuroblastoma cells were grown in Dulbecco’s modified Eagle’s medium (DMEM) supplemented with 10% FBS, 2 mmol L-glutamine, 1% penicillin/streptomycin. The HEK-293 TLR4/MD2/CD14 cells were cultured in DMEM media supplemented with 10% FBS and 1% Penicillin/Streptomycin, 100 µg/mL Hygromycin B and 10 µg/mL Blasticidin. All cells were incubated in a 37°C, 5% CO_2_ humidified atmosphere.

Mouse peritoneal macrophages were isolated by inoculating with 300 *μ*L of sterile 3% sodium thioglycolate (Millipore #1.06691). Three days post-inoculation, animals were euthanized and macrophages were harvested by washing their peritoneal cavity with 10 mL ice-cold PBS. The cell suspension was centrifuged at 500 g for 5 min at 4°C, and the supernatant was discarded. The cell pellet was diluted in RBC lysis buffer (5 mL/mouse) and incubated at room temperature for 10 min, and the supernatant was removed through centrifugation. The cell pellet was suspended in RPMI 1640 media and allowed to attach to the cell culture plate overnight.

Respective cells were added to a density of 1X10^5^ per ml of the cell culture media in 6 well plates. Cells were allowed to adhere and grow overnight followed by serum starvation for 2 Hrs. The adherent cells were treated with the compounds at 37°C for the desired time duration (lysozyme, HMGB1 and LPS; 2 Hrs, PEPinh MyD, PEPinh TRIF, Cu-CPT22, and TAK-242; 4 Hrs and Dynasore; 30 min). Cells were lysed with Cell lytic buffer (Sigma #C2978) with protease inhibitor (Sigma #P4380). Protein samples were normalized for total protein content and western blotting was performed as described previously (19). All the blots were normalized with the internal loading controls and fold change was determined with respect to control samples (placebo treated, or sham-operated) as applicable.

For protein isolation from the animal foot paw, the glabrous foot paw tissue was isolated and lysed in ice-cold tissue lytic buffer with protease inhibitors at 4°C. Rest all the procedures were followed the same as described above.

### Mouse inflammatory cytokine ELISA

Foot pads of hind paws of 6-8 week old mice were injected either with LPS (10µg/50µl) or lysozyme (100µg/50µl). 30 minutes later paw tissue was harvested and rapidly frozen in liquid N_2_ and pulverized. The pulverized tissue was reconstituted in PBS (10% weight by volume). The levels of inflammatory cytokines were measured using ELISA kits according to the manufacturer’s protocol. IFN-α and IL-10 kits were purchased from Thermo and IFN-β from PBL Assay Science. The absorbance (450 nm) was determined using an ELISA plate reader.

### Glutamate assay

The concentration of the neuronal glutamate was estimated using a glutamate assay kit. Briefly, SH-SY5Y cells (1X10^5^ per ml) were plated into two groups. These cells were maintained in DMEM media without glutamate for 4 Hrs followed by treatment with PBS as a control to first group and lysozyme for 2 Hrs to the other group. After the treatment, cells were lysed and lysates were passed through 3kDa membrane filters (Amicon) using centrifugation method. The lysates were used for glutamate estimation. The assay was performed according to the manufacturer’s instructions. Glutamate release in lysozyme-treated cells was compared with the control (PBS-treated) cells.

### Statistical analyses

All the experiments were repeated at least three times. All behavioral data is provided as means standard deviation. Individuals of similar age, weight, and sex were grouped at random, with 6 to 18 animals in each group. GraphPad Prism 9 was used for statistical analysis (GraphPad Software Inc.). A two-way ANOVA was used to examine the behavioral data, followed by a Bonferroni post-test for multiple comparisons. Unless otherwise stated, western blots were evaluated using an unpaired student’s *t* test (two-tailed). For multiple comparisons, all data were analysed using univariate ANOVA or ANOVA for repeated measures, followed by a post-test. All tests were considered significant if the *p*-value was less than 0.05.

## Results

### Lysozyme induces pain without inducing inflammation in mice foot paw models

**Figure 1:**
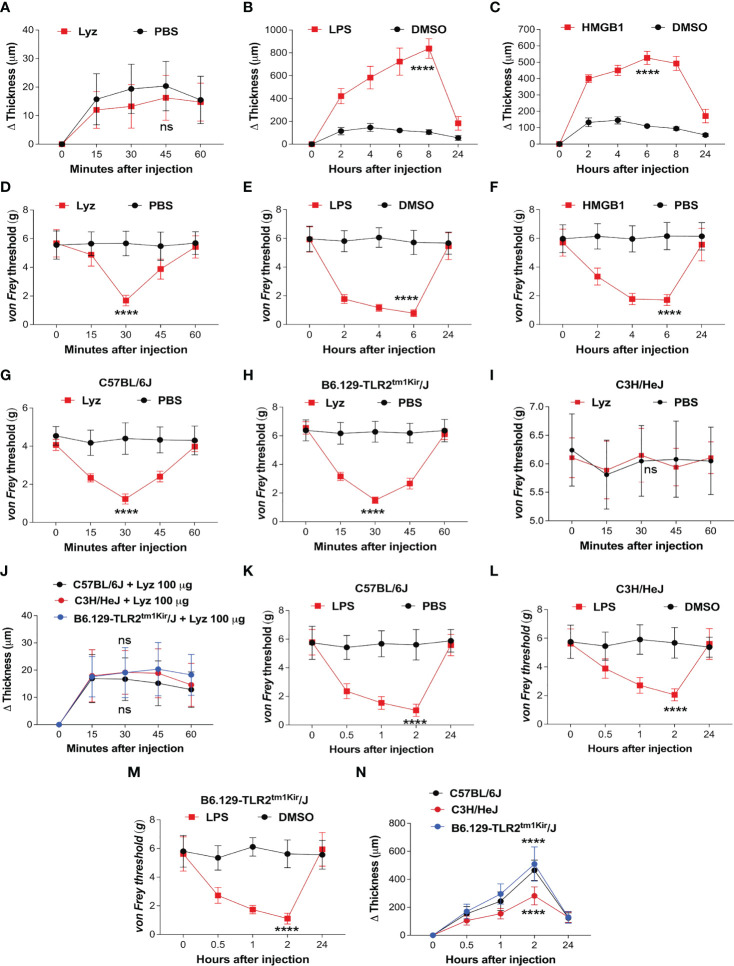
Lysozyme induces pain without inflammation. **(A–C)** Effect of lysozyme **(A)**, LPS **(B)** and HMGB1 **(C)** injections on mouse foot paw inflammation. **(D–F)** Effect of lysozyme **(D)**, LPS **(E)** and HMGB1 **(F)** injections on mouse foot paw pain sensitization. **(G–I)** Effect of lysozyme injections on mice foot paw pain sensitization in **(G)** C57BL/6J **(H)** C3H/HeJ **(I)** B6.129-Tlr2^tm1Kir^/J. (**Figure 1A–I**, n=8-16 animals per group, two-way ANOVA, Tukey’s multiple comparison, *p*<0.001). **(J)** Effect of lysozyme injections on foot paw inflammation in C57BL/6J, C3H/HeJ and B6.129-Tlr2^tm1Kir^/J mice (n=12 animals per group, two-way ANOVA, Tukey’s multiple comparison *p*<0.001). **(K–M)** Effect of LPS injections on mice foot paw pain sensitization in C57BL/6J **(K)**, C3H/HeJ **(L)**, and B6.129-Tlr2^tm1Kir^/J mice **(M)**. (**Figure 1K–M**, n=12 animals per group, two-way ANOVA, Tukey’s multiple comparison, *p*<0.001) **(N)** Effect of LPS injections on foot paw inflammation in C57BL/6J, C3H/HeJ and B6.129-Tlr2^tm1Kir^/J mice (n=12 animals per group, two-way ANOVA, Tukey’s multiple comparison *p*<0.001). ****p> 0.0001; ns, not significant.

TLR4 activation by PAMPs such as bacterial lipopolysaccharide (LPS) or DAMPs like high mobility group box-1 (HMGB1) provokes an intense inflammatory response (20, 21). In our previous study, we identified that lysozyme induces pain through activation of neuronal TLR4. We also observed that unlike LPS, lysozyme-induced pain response was short lived. In the same study we also identified that the mechanism of lysozyme-TLR4 interaction could be different from LPS-TLR4 interaction (19). This led us to investigate how lysozyme injections affect the inflammatory response in mice. We assessed inflammation induced by lysozyme (100 µg/animal) in foot paw (subcutaneous intraplantar injections) of C57BL/6J mice. Interestingly, lysozyme injections did not induce any significant difference in the tissue thickness of the foot paw, indicating no inflammation in these mice (**Figure 1A**) as compared to PBS-injected littermates. In contrast, injections of both LPS (10 µg/animal) and HMGB1 (100 µg/animal) increased the foot paw thickness by 8-fold and 4-fold respectively, indicating a robust inflammatory response in C57BL/6J mice (**Figures 1B, C**). Although interesting, these observations raise a possibility that the dosage of lysozyme used for foot paw injections in the mice is insufficient for it to be effective. This led us to test whether foot paw injections of lysozyme in these mice evoke a pain response. Intraplantar lysozyme (100 µg/animal) injections induced a robust pain response in the ipsilateral foot as compared to PBS-injected mice (**Figure 1D**). Likewise, both LPS (10 µg/animal) and HMGB1 (100 µg/animal) induced pain in C57BL/6J mice (**Figures 1E, F**). We also tested the inflammation induced by a range of lysozyme concentrations effective in inducing pain in these animals. Fascinatingly, none of the dosages (from 100 μg/animal and up to 10-fold more) induced significant inflammation in these mice (**Figure S1A** in **Supplementary Material**), yet, all these dosages induced pain in the mice (**Figure S1B** in **Supplementary Material**). These results show that lysozyme induces pain but not inflammation in the mice foot paw.

Although we have shown that lysozyme induces pain by activation of neuronal TLR4, our findings in the mice foot paw led us to ask if the pain-inducing effects of lysozyme, in the periphery of the body, is TLR4-dependent (19). To test this, we injected lysozyme in wild-type C57BL/6J, C3H/HeJ (TLR4 inactive mutant) mice and B6.129-Tlr2^tm1Kir^/J (TLR2 knockout). Lysozyme injections (100 µg/animal) induced pain in C57BL/6J (**Figure 1G**) and B6.129-Tlr2^tm1Kir^/J (**Figure 1H**) mice groups but not in C3H/HeJ (**Figure 1I**) mice. Interestingly, lysozyme injections did not induce any significant inflammation in any of these mice strains (**Figure 1J**). In contrast, LPS injections (10 µg/animal) in the foot paw of these animals induced both, pain (**Figures 1K–M**) and a robust inflammatory response (**Figure 1N**) in C57BL/6J and B6.129-Tlr2^tm1Kir^/J mice and a milder pain and inflammatory response in C3H/HeJ mice. These results show that unlike LPS, injection of lysozyme does not invoke a strong inflammatory response in mice. To further ascertain this, we performed Evans Blue (EB) assay in both lysozyme and LPS -treated mice groups with PBS-treated mice as control group. In line with our previous experiments, lysozyme-injected mice did not exhibit a strong inflammatory response in comparison to the LPS-treated mice (**Figure S1C** in **Supplementary Material**). Overall, our results indicate that peripheral injections of lysozyme induce pain in a TLR4-dependent manner, however, unlike other TLR4 activators such as LPS, it does not stimulate a significant inflammatory response (20–22).

### Lysozyme activates TLR4 in both neuronal and immune cells

**Figure 2:**
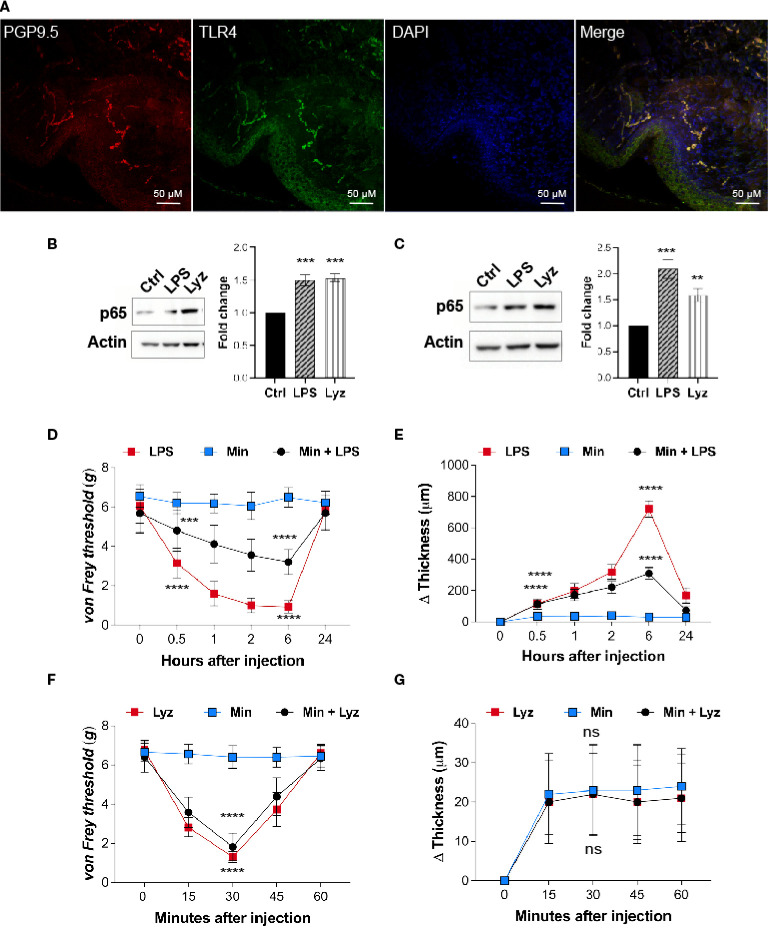
Lysozyme activates TLR4 in neuronal as well as immune cells. **(A)** Representative immunofluorescence micrograph of mouse foot paw sections showing localizations of TLR4 (green) with peripheral neuron marker PGP9.5 (red) (n=3 mice). **(B, C)** Representative western blot images showing expression of p65 upon treatment with lysozyme and LPS in **(B)** mouse peritoneal macrophages and **(C)** THP1 macrophages (**B, C**, n=4 independent experiments, ordinary one-way ANOVA, *p*<0.005). **(D)** Effect of LPS injections on mouse foot paw pain sensitization in presence of minocycline (1 μM) (n=12-16 mice per group, two-way ANOVA, Tukey’s multiple comparison *p*<0.001). **(E)** Effect of LPS injections on mouse foot paw inflammation in presence of minocycline (n=10 mice per group mice, two-way ANOVA, Tukey’s multiple comparison *p*<0.001). **(F)** Effect of lysozyme injections on mouse foot paw pain sensitization in presence of minocycline (n=12 mice per group, two-way ANOVA, Tukey’s multiple comparison *p*<0.001). **(G)** Effect of lysozyme injections on mouse foot paw inflammation in presence of minocycline. (n=10 animals per group, two-way ANOVA, Tukey’s multiple comparison *p*<0.001). The scale bar in the micrograph represents 50 µm.

Lysozyme induces pain by activating neuronal TLR4, hence, we examined if nerve terminals in the mice foot paw express TLR4. Immunofluorescence images from the foot paw tissue sections of C57BL/6J mice showed expression of TLR4 on the nerve terminals in these tissues (**Figure 2A**). PGP 9.5 was used as a marker for the peripheral nerves. Multiple cell types contribute to the pathophysiology of neuropathic pain. TLR4 activators such as LPS and HMGB1 activate macrophage/microglial TLR4 resulting in secretion of pro-inflammatory cytokines which lead to inflammation and pain. However, we found that lysozyme-induced pain without inflammation in a TLR4-dependent manner. This led us to investigate whether there is a cell type selectivity in lysozyme-mediated activation of TLR4 signaling. To test whether lysozyme activates TLR4 in macrophage cells, we treated both mice peritoneal macrophages and differentiated human THP1 macrophages with lysozyme (100 µg/ml of culture media for 2 Hrs) and LPS (10 µg/ml of culture media for 2 Hrs). Both lysozyme and LPS induced NFκB p65 expression in mouse peritoneal macrophage cells (**Figure 2B**) and THP1 macrophage cells (**Figure 2C**) in TLR4-dependent manner. Using NFκB p65 as a readout, we further examined the TLR4 activation in HEK-293 TLR4/MD2/CD14 cells (another cell type). Lysozyme treatment (100 µg/ml of culture media for 2 Hrs) induced NFκB p65 expression in HEK-293 TLR4/MD2/CD14 cells (**Figure S2** in **Supplementary Material**). These observations rule out a cell type specific effect of lysozyme in its activation of TLR4.

Next, we tested whether, akin to LPS, lysozyme-induced pain in the mice foot paw involves macrophage TLR4 activation. To assess the role of macrophages in lysozyme-induced pain in mice foot paw, we injected minocycline, an inhibitor of macrophage activation, in mice foot paw prior to lysozyme or LPS treatment in C57BL/6J mice. Minocycline injections (1μM) reduced LPS (10 µg/animal) induced pain (**Figure 2D**) and inflammation (**Figure 2E**) in mice. However, it did not affect lysozyme (100 µg/animal) induced pain (**Figure 2F**) and had no effect on inflammation in any way (**Figure 2G**). These results confirm that lysozyme could activate macrophage TLR4, and unlike LPS, pain induction by lysozyme is not mediated by macrophage activation. Collectively, our findings show that although TLR4 activation by lysozyme is not cell selective, yet induction of pain by it may be independent of the inflammatory mediators secreted from the activated macrophages.

### Lysozyme selectively activates TRIF pathway through TLR4

**Figure 3:**
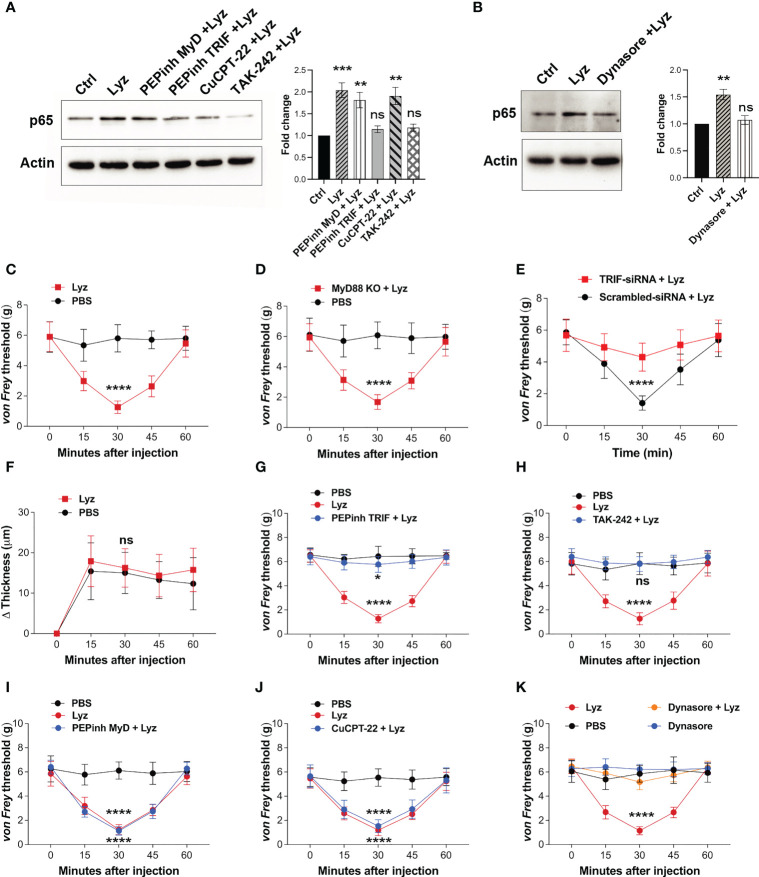
Lysozyme exerts its effects through TRIF activation. **(A, B)** Western blot images showing effect of TRIF inhibition on NFκB expression in SH-SY5Y cells **(A)** effect of specific MyD and TRIF inhibitors on lysozyme-mediated expression of NFκB p65 **(B)** effect of internalization inhibitor Dynasore on NFκB p65 expression (n=4 independent experiments, ordinary one-way ANOVA, *p*<0.005). **(C–E)** Effects of lysozyme injections on pain sensitization on C57BL/6J **(C)**, MyD88 Knockout **(D)**, and TRIF-siRNA-treated healthy mice **(E)**. **(F)** Effects of lysozyme injections on inflammation in TRIF-siRNA-treated mice foot paw. (**Figures 3C–F**, n=8-12 animals per group, two-way ANOVA, Tukey’s multiple comparison *p*<0.001). **(G–J)** Effects of intracellular signaling inhibitors; PEPinhMyD **(G)**, PEPinhTRIF **(H)**, TAK-242 **(I)** and CuCPT-22 **(J)** on pain sensitization by lysozyme. **(K)** Effect of internalization inhibitor Dynasore on lysozyme-mediated pain sensitization. (**Figures 3G–K**, n=8-12 animals, two-way ANOVA followed by Tukey’s multiple comparison *p*<0.001). *p>0.0021; ***p>0.0002; ****p>0.0001; ns, not significant.

Pain sensitization by lysozyme in mice foot paw is TLR4-dependent. It also activates macrophage TLR4, yet, lysozyme injections do not induce inflammation. This prompted us to investigate the activation of the intracellular signaling upon TLR4 activation. Its activation by LPS stimulates both MyD88-dependent and -independent (TRIF-dependent) pathways which ultimately lead to expression and secretion of various pro and anti-inflammatory cytokines and chemokines (21). We treated human neuronal SH-SY5Y cells with PEPinh MyD (MyD88 inhibitor) (100 µM), PEPinh TRIF (TRIF inhibitor) (100 µM), TAK-242 (TLR4 inhibitor) (1 µM), and CuCPT-22 (TLR2 inhibitor) (1 µM) followed by treatment with lysozyme (100 µg/ml of culture media for 2 Hrs). Both PEPinh TRIF as well as TAK-242 inhibited lysozyme-induced NFκB p65 activation, whereas CuCPT-22 had no effect. Interestingly, PEPinh MyD did not alter lysozyme-mediated NFκB p65 activation (**Figure 3A**). This result suggests that TLR4 activation by lysozyme triggers TRIF signaling and not MyD88-dependent signaling. TRIF signaling activation takes place in the endosomes after TLR4 internalization. Hence, the inhibition of endocytosis may lead to inhibition of TRIF activation by lysozyme. We treated SH-SY5Y cells with Dynasore (an endocytosis inhibitor) for 30 min followed by lysozyme treatment (100 µg/ml of culture media for 2 Hrs). Dynasore (80 μM) treatment abrogated TLR4-NFκB p65 activation by lysozyme (**Figure 3B**), thus corroborating our finding that TLR4 activation by lysozyme invokes TRIF signaling.

Next, we tested whether selective activation of TRIF signaling by lysozyme is responsible for its pain-inducing effects. We injected lysozyme in C57BL/6J, MyD88 knockout and C57BL/6J TRIF knockdown (treated with siRNA against TRIF) mice groups. Lysozyme injections in C57BL/6J, and MyD88 knockout mice groups induced pain response in these animals (**Figures 3C, D**). However, mice treated with TRIF-siRNA (intrathecal, 400 μg/animal) did not exhibit pain response upon lysozyme injections (**Figures 3E**, **S3** in **Supplementary Material**). In line with the previous experiments, lysozyme injections in these mice did not induce inflammation (**Figure 3F**). To further confirm these findings, we injected PEPinh MyD (1 mM), PEPinh TRIF (1 mM), TAK-242 (10 µM) and CuCPT-22 (10 µM) in C57BL/6J mice groups, followed by lysozyme (100 µg/animal) injections. While both PEPinh TRIF and TAK-242 abrogated lysozyme-induced pain (**Figures 3G, H**), PEPinh MyD and CuCPT-22 did not do so (**Figures 3I, J**). Further, treatment of mice with Dynasore, prior to lysozyme treatment, abrogated pain (**Figure 3K**). Overall, our results reveal that lysozyme selectively activates MyD88-independent TRIF signaling through TLR4 activation. Specific activation of TRIF signaling by lysozyme results in pain sensitization without any significant inflammatory response.

### TLR4 activation by lysozyme does not induce robust inflammatory cytokine response

**Figure 4:**
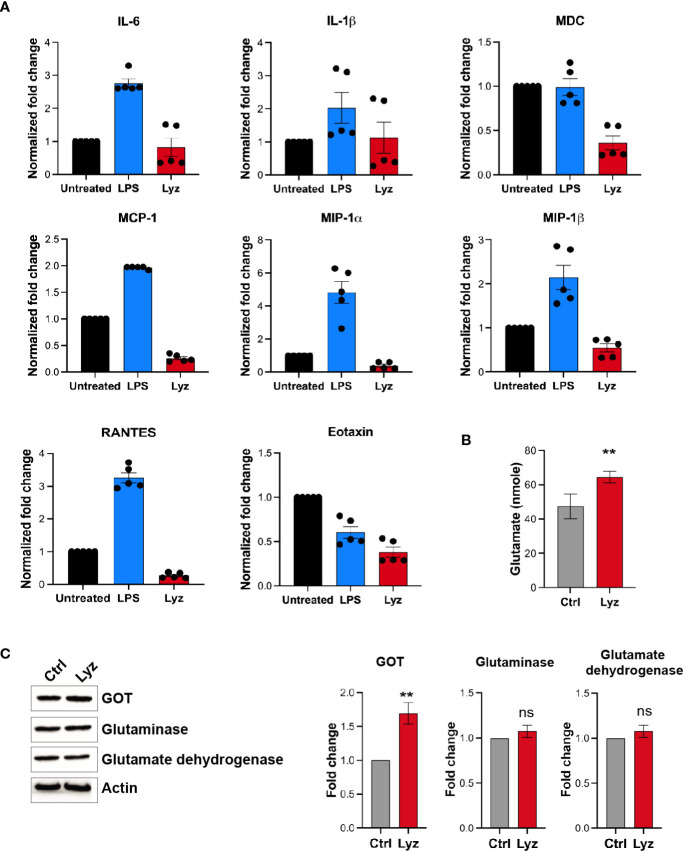
Lysozyme induces pain in absence of a robust inflammatory cytokine response. **(A)** Comparative analysis of lysozyme and LPS mediated regulation of inflammatory cytokines (n=4 animals, multiple *t*-tests *p*<0.05 at 30 min). **(B)** Effects of lysozyme treatment on neuronal glutamate amount in SH-SY5Y cells (n=4 independent experiments, *p*<0.005 versus Lyz treatment, ordinary one-way ANOVA). **(C)** Lysozyme treatment changes the expression of glutamate oxaloacetate transaminase (GOT) in SH-SY5Y cells (n=4 independent experiments, ordinary one-way ANOVA, *p*=0.005 versus Lyz treatment) All western blots repeats were performed with different tissue samples and analysed using unpaired (two-tailed) *t*-test. ns,not significant.

Activation of TLRs during infection and injury leads to an inflammatory response. However, lysozyme injections induced pain but no inflammation by selective activation of TRIF signaling through TLR4. To gain mechanistic insight into lysozyme-induced pain without inflammation, we performed a comparative profiling of pro-inflammatory cytokines and chemokines expression post- lysozyme and LPS injections. Groups of C57BL/6J mice were injected with lysozyme (100 µg/animal), LPS (10 µg/animal) and a control group were injected with DMSO. Inflammatory cytokines were measured from the mice foot paw tissue lysates of the respective groups after 30 minutes of injections. LPS injections increased the production of pro-inflammatory cytokines including Interleukins IL1β, IL6, RANTES, and chemokines such as MCP1, MIP-1α, MIP-1β were significantly up-regulated as compared with the control group. However, some other cytokines and chemokines like Tumour necrosis factor (TNF), IL17A, IL12, MDC, SDF-1, and Eotaxin did not change significantly (**Figure 4A**, **S4** in **Supplementary Material**). Interestingly, lysozyme treatment in mice foot paw resulted in a blunted response in the pro-inflammatory cytokines and chemokines as compared to that of LPS. Lysozyme-treated mice had significantly different cytokines profile where IL1β and IL6 showed markedly lower expression as compared to LPS-injected mice. TNF, IL12, MIP-1α, and MIP-1β concentrations were unaffected and SDF-1, RANTES, MCP1, MDC and Eotaxin were downregulated as compared with the control mice (**Figure 4A**). This finding confirmed that TLR4 activation by lysozyme induces negligible inflammatory cytokine response. Furthermore, the blunted cytokine profile upon lysozyme treatment was different from that of LPS-induced cytokine flux. Activation of TRIF pathway by TLR4 induces type I IFN response as well as it could also induce activation of anti-inflammatory IL-10 response (23). We checked the expression of IFNα/β and IL-10 in response to LPS and lysozyme treatments in mice groups. Both LPS and lysozyme treatment enhanced the expression of IFNα and IFNβ with respect to PBS-treated control group (**Figure S4** in **Supplementary Material**). Interestingly, IL-10 expression was enhanced in the lysozyme-treated group but not in the LPS-treated group (**Figure S4** in **Supplementary Material**). These results corroborate further that lysozyme activates TRIF signaling through TLR4.

LPS induces pain through enhanced pro-inflammatory cytokines. These cytokines then act on the neurons through their respective receptors and enhance pain sensitivity. Although, lysozyme injections in mice foot paw induced pain in a TLR4-dependent manner, unlike LPS it did not induce a robust inflammatory cytokine flux. This led us to explore the molecular mechanism leading to the lysozyme-mediated pain sensitization in neurons.

### Lysozyme treatment induces neuronal glutamate

Our results show that activation of TLR4 by lysozyme invokes TRIF signaling, which results in pain sensitization without inducing a robust proinflammatory cytokine response. This leads to the question if it’s not the proinflammatory cytokines then what causes enhanced neuronal hyperexcitability upon lysozyme treatment? Glutamate is the principal excitatory neurotransmitter which is involved in the sensory neuronal hyperexcitability during nerve injury (24, 25). Also, neuronal TLR4 activation induces AMPA currents (26) leading to neuronal hyperexcitability during brain injuries and AMPA currents are mediated by glutaminergic neurotransmission. Hence, we tested whether lysozyme treatment increases glutamate in neuronal cell culture. Human neuronal SH-SY5Y cells were treated with lysozyme (100 μg/ml of culture media for 2 Hrs) or PBS control, and glutamate concentrations were quantified in these neuronal cultures. Glutamate amount were increased in the cells treated with lysozyme as compared with the PBS-treated cells (**Figure 4B**) from 47 nmoles in control group to 63 nmoles in lysozyme-treated group. Thus, lysozyme action on neuronal cells is sufficient to induce glutamate in neurons.

We investigated for the molecular mediators leading to enhanced glutamate in the neurons upon lysozyme treatment. In mammalian cells, glutamate is synthesized majorly by three enzymes, Glutamate dehydrogenase, Glutaminase and Glutamate oxaloacetate transaminase (GOT). Thus, we probed for the expression of these enzymes in SH-SY5Y cells post lysozyme (100 μg/ml of culture media for 2 Hrs) treatment. Western blotting experiments showed that lysozyme treatment did not affect the expression of glutamate dehydrogenase and glutaminase, however, we observed significant enhanced expression of GOT (by 1.7-fold) from lysozyme-treated cells as compared with the PBS-treated cells (**Figure 4C**). These results indicate that lysozyme could enhance neuronal glutamate in absence of proinflammatory cytokines.

### TLR4 activation by lysozyme is required for GOT overexpression

**Figure 5:**
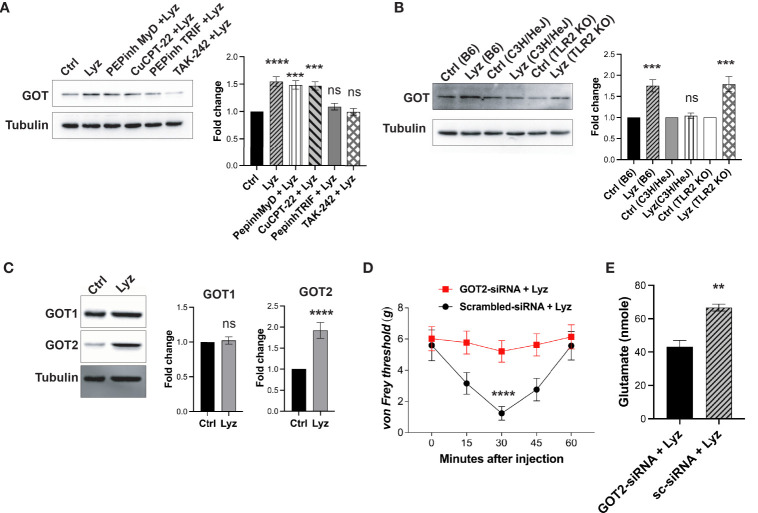
Lysozyme induces GOT overexpression by TLR4 activation. **(A, B)** Representative western blot images showing effects of lysozyme-mediated activation of TLR4 on GOT expression in **(A)** SH-SY5Y human neuronal cells treated with lysozyme (n=4 independent experiments, ordinary one-way ANOVA followed by Dunnett’s multiple comparisons test *p*<0.005) **(B)** mice treated with lysozyme (n=4 independent experiments, ordinary one-way ANOVA *p*<0.005). **(C)** Representative western blot images showing the effects of lysozyme on the expression of two different GOT isoforms in SH-SY5Y cells (n=4 independent experiments, *t*-tests *p*<0.005). **(D)** Specific inhibition of GOT2 isoform affects lysozyme-mediated pain sensitization in mice (n=10 mice per group, two-way ANOVA followed by Sidak’s multiple comparison *p*<0.005). **(E)** Effect of lysozyme treatment on neuronal glutamate amount in GOT2-siRNA-treated SH-SY5Y cells (n=4 independent experiments, *p*<0.005 versus Sc-siRNA treatment, ordinary one-way ANOVA).

We next tested whether lysozyme-mediated GOT overexpression is TLR4- dependent or independent. We treated SH-SY5Y neuronal cells with PEPinh MyD (100 µM), PEPinh TRIF (100 µM), TAK-242 (1 µM) and CuCPT-22 (1 µM) prior to treatment with lysozyme (100 μg/ml of culture media for 2 Hrs). Pre-treatment of these cells with selective intracellular signaling inhibitor PEPinh TRIF as well as selective TLR4 inhibitor TAK-242 did not result in any change in GOT expression upon lysozyme treatment. On the contrary, lysozyme treatment in PEPinh MyD and TLR2 specific inhibitor Cu-CPT22 treated cells resulted in overexpression of GOT in these cells (**Figure 5A**). Further, C57BL/6J, C3H/HeJ (TLR4 inactive mutant) and B6.129-Tlr2^tm1Kir^/J (TLR2 knockout) mice were injected with lysozyme (100 μg/animal) in their foot paw. Lysozyme injections in C3H/HeJ had no effect on GOT expression, however, lysozyme injections in C57BL/6J and B6.129-Tlr2^tm1Kir^/J mice resulted in enhanced GOT (by 1.6-fold) expression in the foot paw of these mice (**Figure 5B**). Thus, lysozyme-mediated TLR4 activation results in overexpression of GOT. In mammalian cells, two isoforms of GOT are expressed, a cytosolic GOT1 and mitochondrial GOT2 (17). We probed for the GOT isoform being induced by lysozyme. In SH-SY5Y neuronal cells treated with lysozyme (100 μg/ml of culture media for 2 Hrs), GOT2 expression was enhanced significantly, however, GOT1 expression remained unaffected (**Figure 5C**). This led us to test whether GOT2 overexpression leads to lysozyme-mediated pain sensitization. We injected GOT2-siRNA (intrathecal, 400 μg/animal) into C57BL/6J mice for 3 days prior to lysozyme (100 μg/animal) injection in foot paw. Another group of mice were injected with scrambled-siRNA (Intrathecal, 400 μg/animal) followed by lysozyme (100 μg/animal) injections. GOT2 siRNA injections mitigated lysozyme-induced pain sensitization as compared with scrambled siRNA-treated mice (**Figure 5D**). Subsequently, we also tested whether lysozyme-induced overexpression of GOT2 results in heightened glutamate build-up in neuronal cells. SH-SY5Y neuronal cells were treated with GOT2-siRNA. Subsequent treatment with lysozyme (100 μg/ml of culture media for 2 Hrs) in these cells had no change in the glutamate concentrations (from 43 nmoles), whereas, lysozyme treatment in neuronal cells pre-treated with scrambled siRNA led to increased glutamate concentrations from 43 nmoles to 66 nmoles (**Figures 5E**, **S5** in **Supplementary Material**). Collectively, these results indicate that lysozyme-mediated TLR4 activation in neurons leads to enhanced expression of GOT2 which leads to heightened pain sensitization in a glutamate dependent manner.

## Discussion

Our previous study revealed that lysozyme up-regulation during nerve injury activates neuronal TLR4 leading to neuropathic pain (19). TLR4 activation in cells of immune system as well as glial cells of the nervous system induces MyD88 and TRIF signaling, thus activating transcription factors (viz. NFκB, IRF etc.) (21). This results in overexpression and secretion of various pro and anti-inflammatory cytokines. These cytokines impart distinct physiological outcomes on the local inflammatory milieu. However, very little is known about the effects of neuronal TLR4 activation and how it influences neuroinflammation. In the present study, we investigated the effects of neuronal TLR4 activation by lysozyme on both inflammation and pain sensitization. Inflammation in the mice foot paw can be measured accurately, thus, we injected lysozyme in mice foot paw to assess the inflammation as a result of TLR4 activation. Intriguingly, lysozyme injections did not induce inflammation in mice foot paw as compared with other TLR4 activators such as LPS. Yet, it induced significant pain response in mice ipsilateral foot paw. The pain inducing effects of lysozyme were short-lived as compared with the LPS. This can be because of the short stability of mammalian lysozyme *in-vivo*. This is in the order of minutes in rodents (about 75 min) (27). Since lysozyme-induced pain effects are short-lived (peak in 30 min), we thought that short lived effects of lysozyme may be the reason behind the insignificant inflammatory response. However, LPS induced significant inflammatory response in 30 minutes which ruled out this possibility. We also observed inflammation in the foot paw of C3H/HeJ mice upon LPS injections. This could be attributed to the inflammation through the activation of non-canonical inflammasome by LPS (28). These findings led us to hypothesize that lysozyme induces pain without inflammation. Consistent with our previous data, we found that lysozyme-induced pain in mice foot paw was TLR4-mediated.

Activation of TLR4 in immune cells leads to a significant immune response which results in an inflammatory response (22). Lysozyme injections in the foot paw did not induce any inflammation. This led us to investigate whether lysozyme activates TLR4 in immune cells. Our results indicate that lysozyme activates TLR4 in mouse macrophages as well as in human macrophage cell lines. Hence, lysozyme-mediated TLR4 activation is not cell type selective. Moreover, the macrophage inhibition (by minocycline) experiments also showed that lysozyme-mediated pain effects were not dependent on macrophage TLR4 activation. Although TLR4 activation by lysozyme is not cell type specific, its pain-inducing effects are independent of immune cell TLR4 activation (such as macrophages) and subsequent inflammatory response. Though these experiments show that lysozyme-mediated pain sensitization is disassociated from immune cells mediated inflammatory response, they do not indicate how lysozyme injections fail to induce a significant inflammatory response. Failure of lysozyme in inducing inflammation despite its activation of TLR4, hence, appears perplexing. To answer this, we probed the intracellular signaling response as a result of TLR4 activation by lysozyme and noted that lysozyme preferentially activates MyD88-independent (TRIF) pathway through TLR4 in SH-SY5Y neuronal cells. There is no known natural agonist of TLR4 which can selectively activate TRIF pathway, in neurons, upon binding with TLR4. Further, we show that the peripheral neurons in the mice foot paw express TLR4, These results in conjunction with our previous study, which shows that TLR4 activation by lysozyme in neurons is sufficient for pain sensitization, point towards a possibility that selective TRIF activation by lysozyme in peripheral nociceptors induces pain. At the same time, it might lead to a weaker inflammatory response (in absence of MyD88 signaling activation) in neurons as well as other cell types, hence, curtailing inflammatory outcomes observed with other TLR4 activators such as LPS. It is important to highlight here that in our previous study we identified that lysozyme interacts with annexin A2 to activate TLR4. This is different from the typical TLR4 activation mechanism (which involves CD14 and MD-2) and it could be the reason behind the specific activation of TRIF pathway by lysozyme. However, further studies will be required to uncover the mechanistic details of this atypical TLR4 activation by lysozyme. TLR4 activation by its endogenous activators and its physiological effects is an active area of research (29, 30), and lysozyme appears to be unique in primarily recruiting TRIF pathway and not the MyD88 pathway, among the natural TLR4 activators.

We attempted to identify the mechanism through which lysozyme can induce neuronal (nociceptor) sensitization in absence of enhanced inflammatory cytokine flux which is known to transduce pain signals through nociceptors (31). Our next set of experiments showed that lysozyme application stimulates neuronal glutamate, thus, hinting that excitatory glutaminergic neurotransmission which is often associated with enhanced pain sensitivity could be enhanced by lysozyme action on neurons. A further dissection of the molecular details indicates that enhanced expression of GOT in neurons by lysozyme application could be the reason behind increased glutamate. We also found that the mitochondrial isoform of GOT (GOT2) was selectively overexpressed upon lysozyme application which could lead to increased glutamate. This argument is intuitive as GOT2 is also expressed in the presynaptic vesicles carrying glutamate (32). Its enhanced expression is shown to increase glutamate packaging in the presynaptic vesicles (33). Thus, any increase in GOT2 expression as a result of lysozyme treatment, could result in enhanced presynaptic glutamate release. siRNA knockdown of GOT2 relieved lysozyme-mediated pain in mice. This indicated a direct role of GOT in lysozyme-mediated pain sensitization. We also showed that TLR4 activation is essential for enhanced expression of GOT2 by lysozyme. Thus, lysozyme increases neuronal excitability through neuronal TLR4, in absence of inflammatory cytokines, by increasing GOT2 expression.

Pain has been classified as a cardinal feature of inflammation and the molecular mechanisms through which inflammatory cytokines activate nociceptors to induce pain during inflammation have been probed in considerable detail. However, later it was shown that mechanism by which pain is induced during bacterial infection could be independent of inflammation (11). Non-steroid anti-inflammatory drugs (NSAIDs) exhibit mixed results in eliminating pain during many painful conditions such as neuropathic pain (34). This indicates independence of the axis of pain from inflammation. Thus, the inflammation independent mechanisms of pain could be the reason behind failure of NSAIDs in mitigating pain in a large number of patients with chronic pain. Despite being significant component of chronic pain, very limited information is available about the inflammation independent mechanisms of pain transduction. Moreover, in chronic pain, pain often persists after resolution of inflammation and hence it becomes critical to understand the mechanisms of pain in absence of inflammatory mediators, which may aid in designing novel drugs against chronic pain. Our investigation uncovers an important aspect of pain transduction during sterile inflammation. We show that activation of neuronal TLR4 by lysozyme induces pain in absence of concomitant pathologic inflammation. Multiple studies have shown the expression of TLR4 on both central and peripheral neurons. Yet, our understanding about the role of these receptors in neuronal pathophysiology is very limited. Our present study uncovers a mechanism by which activation of these receptors in neuronal cells can alter the neuronal excitability without manifesting overt inflammation. Our previous study (19) showed that during neuronal injury, lysozyme is overexpressed in the neurons and contributes in the pathophysiology of neuropathic pain.

Thus, our present study together with our previous study shows that during neuronal injury, a continuous release of neuronal lysozyme can activate neuronal TLR4. Lysozyme-activated TLR4 recruits TRIF axis for cytokine activation which invoke pain by activating the nociceptors while avoiding the damaging consequences of severe inflammation. In contrast to other TLR4 agonists such as LPS, HMGB1 etc., which induce a strong inflammatory response and pain, lysozyme can lead to pain sensitization in absence of inflammation. Lysozyme recruits Annexin A2 to activate TLR4-TRIF axis of signaling, leading to a couple of possibilities for future investigations; (1) is lysozyme a biased agonist viz. is it a direct ligand? or (2) an indirect activator requiring a mediator such as Annexin A2. Further, these findings about the ability of lysozyme to evoke pain without inflammation opens two possibilities; (1) does this mechanism aid in transition of acute pain to chronic pain (which often outlasts the inflammatory response post injury); (2) induction of pain by lysozyme without inflammation together with an expression of an anti-inflammatory cytokine (i.e., IL-10), perhaps aids in healing and repair post injury. Further studies will be required to uncover the details of these possibilities.

## Data availability statement

The original contributions presented in the study are included in the article/**Supplementary Material**. Further inquiries can be directed to the corresponding author.

## Ethics statement

The animal study was reviewed and approved by the animal ethics committee, Indian Institute of Science.

## Author contributions

AvS and SY conceived the study. SY designed the study and performed the experiments. AmS helped in experiments. RK performed EB assay and the type I IFN and IL-10 ELISA. SY and AmS analysed data. SY, AmS. and AvS wrote the manuscript. All authors contributed to the article and approved the submitted version.
