# Supplementary material for: TLR4 activation by lysozyme induces pain without inflammation
Source: Front Immunol. 2023 May 1;14:1065226. doi: 10.3389/fimmu.2023.1065226 (PMC10183575; doi:10.3389/fimmu.2023.1065226)
Supplement: Supplementary file 1 [file DataSheet_1.pdf]

Supplementary Material

TLR4 activation by lysozyme induces pain without inflammation

Saurabh Yadav, Amrita Singh, Ravi Kant, Avadhesha Surolia*

*** Correspondence:** Corresponding Author: surolia@iisc.ac.in

1.1 Supplementary Figures

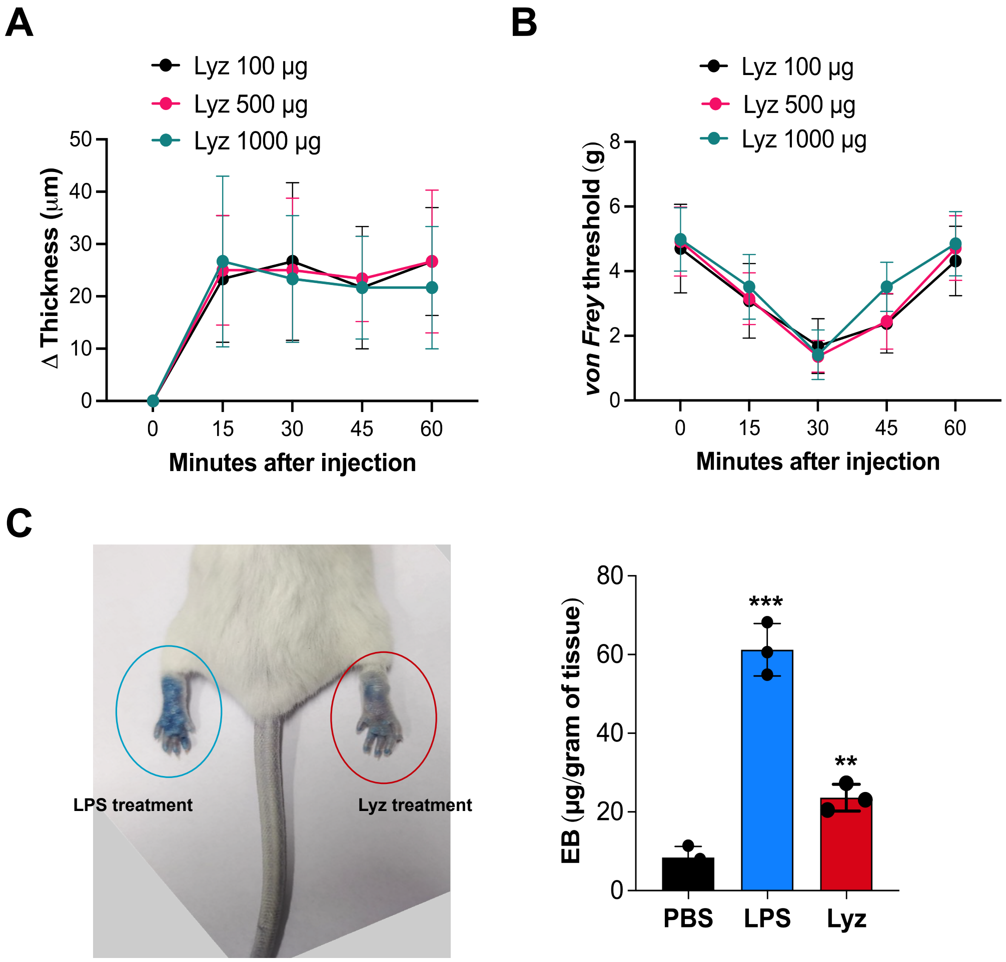

Supplementary Figure S1 | Increasing lysozyme concentrations induce pain but no inflammation. (**A**) Comparative analysis of increasing lysozyme concentrations on inflammation (n=6 animals/group, multiple *t*-tests at 30 min). (**B**) Effects of increasing lysozyme concentrations on pain (n=6 animals/group ordinary one-way ANOVA, $p<0.005$). (**C**) Representative image and bar graph showing the effects of LPS and lysozyme treatment on mouse foot paw inflammation by Evans Blue (EB) assay (n=3 animals per group, ordinary one-way ANOVA $p<0.005$).

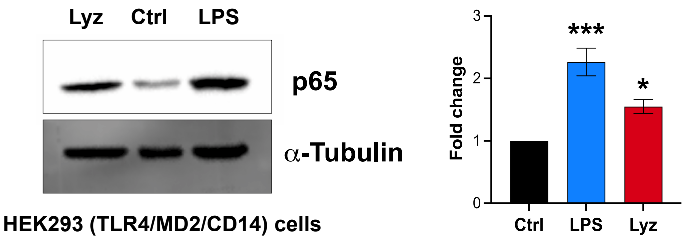

Supplementary Figure S2 | Representative western blot images showing change in expression of NFκB p65 in HEK-293 TLR4/MD2/CD14 cells post lysozyme treatment (n=4 independent experiments).

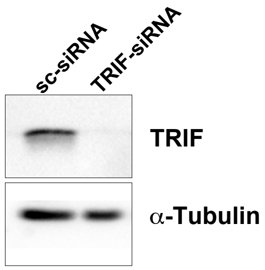

Supplementary Figure S3 | Western blot images showing silencing efficacy of TRIF siRNA with respect to scrambled siRNA (sc-siRNA). siRNAs were injected daily (intrathecal injections of 400 μg/animal) for 3 consecutive days. On day 4 DRG tissue samples were isolated and processed for western blotting.

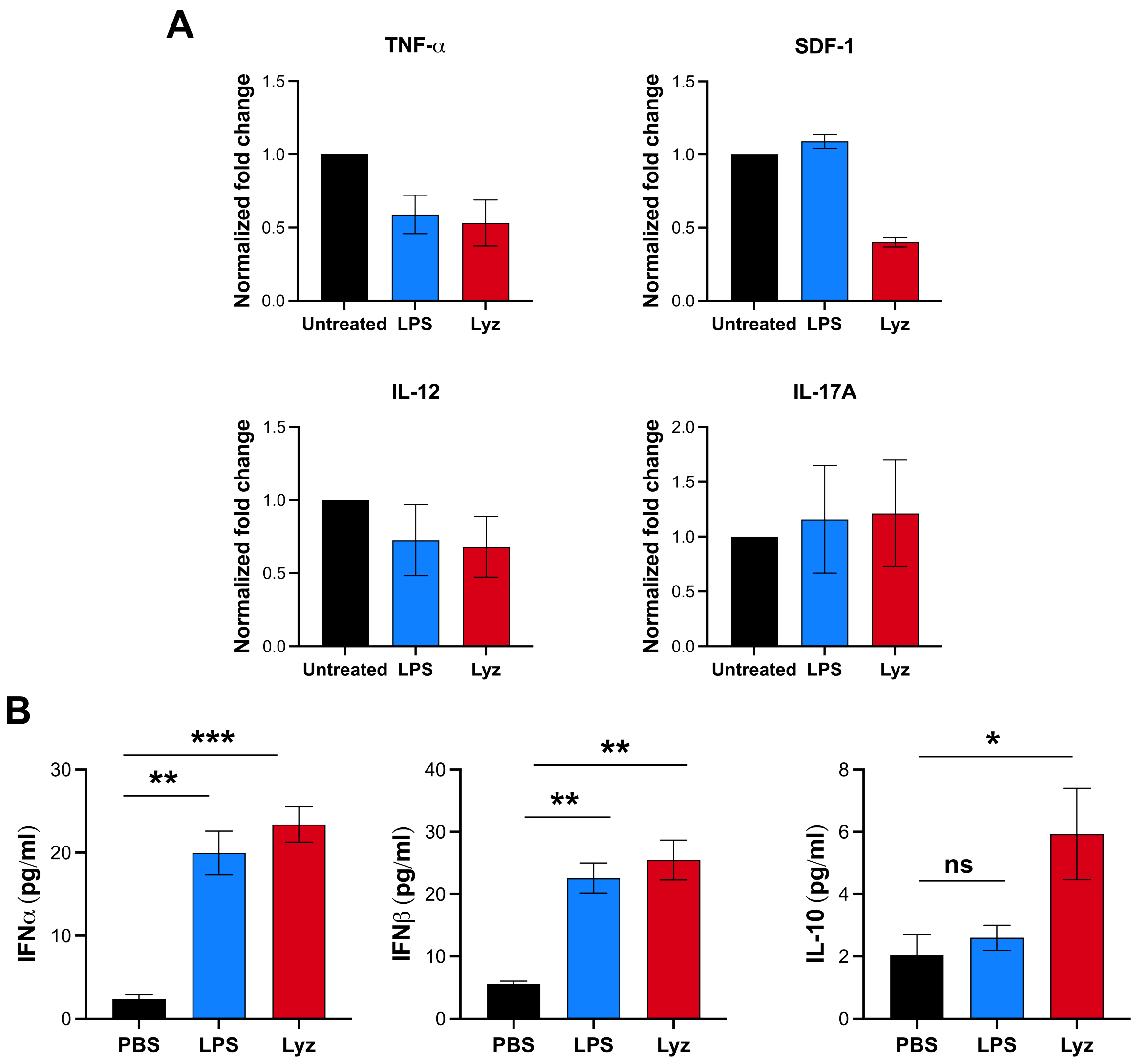

Supplementary Figure S4 | (**A**) Lysozyme induces pain in absence of a robust inflammatory cytokine response. Comparative analysis of lysozyme and LPS mediated regulation of inflammatory cytokines (n=5 animals/group, multiple *t*-test $p<0.05$ at 30 min). (**B**) Bar graphs showing the effects of LPS and lysozyme injections on type I IFNα/β and IL-10 expression in mice foot paw (n=3 animals/group ordinary one-way ANOVA, $p<0.005$).

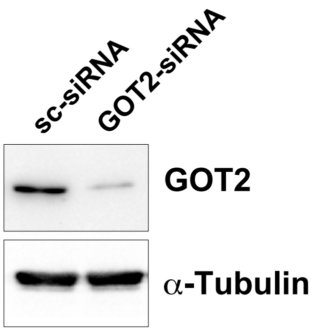

Supplementary Figure S5 | Western blot images showing silencing efficacy of GOT2 siRNA with respect to scrambled siRNA (sc-siRNA). siRNAs were injected daily (intrathecal injections of 400 μg/animal) for 3 consecutive days. On day 4 DRG tissue samples were isolated and processed for western blotting.
